# PKA inhibition is a central step in D,L-methadone-induced ER Ca^2+^ release and subsequent apoptosis in acute lymphoblastic leukemia

**DOI:** 10.3389/fcell.2024.1388745

**Published:** 2024-04-24

**Authors:** Hamza Kamran, Jung Kwon Lee, Ki-Young Lee

**Affiliations:** Department of Cell Biology and Anatomy, Arnie Charbonneau Cancer and Alberta Children’s Hospital Research Institutes, University of Calgary, Calgary, AB, Canada

**Keywords:** signaling, calcium, apoptosis, leukemia, cancers

## Abstract

Acute lymphoblastic leukemia (ALL) is a hematologic cancer that mostly affects children. It accounts for over a quarter of ALL pediatric cancers, causing most of the cancer death among children. Previously, we demonstrated that D,L-methadone causes ALL cell apoptosis via μ-opioid receptor 1 (OPRM1)-triggered ER Ca^2+^ release and decrease in Ca^2+^ efflux, elevating [Ca^2+^]_i_. However, the precise mechanism by which D,L-methadone induces ER Ca^2+^ release remains to be defined. Here, we show that in ALL cells, D,L-methadone-induced ER Ca^2+^ release is blocked by inhibition of G_αi_, but not G_βϒ_, indicating that the process is dependent on G_αi_. Activation of adenylyl cyclase (AC) with forskolin or treatment with 8-CPT-cAMP blocks D,L-methadone-induced ER Ca^2+^ release, indicating that the latter results from G_αi_-dependent downregulation of AC and cAMP. The 14–22 amide (myr) PKA inhibitor alone elicits ER Ca^2+^ release, and subsequent treatment with D,L-methadone does not cause additional ER Ca^2+^ release, indicating that PKA inhibition is a key step in D,L-methadone-induced ER Ca^2+^ release and can bypass the D,L-methadone-OPRM1-AC-cAMP step. This is consistent with the decrease in PKA-dependent (i) inhibitory PLCβ3 Ser1105 phosphorylation that leads to PLCβ3 activation and ER Ca^2+^ release, and (ii) BAD Ser118 phosphorylation, which together ultimately result in caspase activation and apoptosis. Thus, our findings indicate that D,L-methadone-induced ER Ca^2+^ release and subsequent apoptosis in ALL cells is mediated by G_αi_-dependent downregulation of the AC-cAMP-PKA-PLCβ3/BAD pathway. The fact that 14–22 amide (myr) alone effectively kills ALL cells suggests that PKA may be targeted for ALL therapy.

## Introduction

Acute lymphoblastic leukemia (ALL) is the most common malignancy in children that is characterized by rapid expansion of immature lymphocytes in the blood and bone marrow (Capria et al., 2020). The development of ALL has been linked to exposure to environmental hazards such as benzene, ionizing radiation, or previous exposures to chemotherapy and radiotherapy, and mutations in one or more of the genes that control hematopoiesis (Midic et al., 2020). Chemotherapeutic drugs have predominantly been used to target leukemic cells through mechanisms that include double-stranded DNA breaks, inhibition of protein and nucleic acid synthesis, and disruption of mitotic cell division (Terwilliger and Abdul-Hay, 2017). However, leukemic cells often develop resistance to chemotherapy drugs, resulting in poor patient prognosis (Zhang et al., 2019). Thus, the search for improved therapy for ALL is imperative.

D,L-methadone is a synthetic opioid that targets neuronal cells through G-protein-coupled receptors, GPCRs. It has conventionally been used as a pain killer and as a treatment for patients suffering from severe addictions to more potent opioids such as heroine and fentanyl (Ali et al., 2017). Like neuronal cells, leukocytes express opioid receptors (Celik et al., 2016) that when stimulated modulate proliferation, chemotaxis, cytokine production and cytotoxicity (Bidlack, 2000; Sharp, 2006; Ninkovic and Roy, 2013). Stimulation of opioid GPCRs causes alteration in receptor conformation, which allows GDP to GTP exchange on G protein α subunit (G_α_), one of the heterotrimeric G protein complexes (G_αβγ_), resulting in uncoupling of G_α_ from G_βγ_ (Al-Hasani and Bruchas, 2011). Both G_βγ_ dimer and GTP-bound G_α_ are involved in pain modulation (Celik et al., 2016) and apoptosis (Friesen et al., 2013), respectively, by activating their effectors: Ca^2+^ or cyclic adenosine monophosphate (cAMP). Dissociated G_βγ_ stimulates phospholipase C beta (PLCβ) that hydrolyzes phosphatidylinositol-4,5-bisphosphate (PIP2) into inositol 1,4,5-trisphosphate (IP3) and diacylglycerol. IP3 triggers endoplasmic reticulum (ER) Ca^2+^ release via IP3 receptor (IP3R) Ca^2+^ channel, which in leukocytes leads to secretion of opioid peptides such as Met-enkephalin (Labuz et al., 2009), β-endorphin (Labuz et al., 2010), and dynorphin (Liou et al., 2011) which mediate the pain relief response (Celik et al., 2016). Conversely, GTP-bound G_αi_ blocks adenylyl cyclase (AC) activity, resulting in reduced [cAMP]_i_, activation of caspases, and induction of apoptosis (Friesen et al., 2013).

Previously, it was shown that D,L-methadone induces ALL cell apoptosis through stimulation of an opioid receptor, downregulation of cAMP and upregulation of caspases (Friesen et al., 2008; Friesen et al., 2013): opioid receptor-G_αi_-AC-↓[cAMP]_i_-caspase pathway. Since there are at least four different types of opioid receptors and the precise mechanism through which D,L-methadone induces ALL cell apoptosis was not known, we previously investigated these issues and demonstrated that D,L-methadone-induced ALL apoptosis occurred via the μ-opioid receptor 1 (OPRM1), triggering ER Ca^2+^ release and decreased Ca^2+^ efflux, elevating [Ca^2+^]_i_ that upregulated the calpain-1-Bid-cytochrome C-caspase-3/12 apoptotic pathway (Lee et al., 2021): OPRM1-↑[Ca^2+^]_i_-calpain-1-Bid-cyt C-caspase-3/12 pathway. Since cAMP activates protein kinase A (PKA), which phosphorylates (i) PLCβ3 at Ser1105, inhibiting PLCβ3 activation (Yue et al., 1998), and (ii) BAD at Ser118, disrupting BCL-2-BAD interaction, which stimulates the BCL-2 anti-apoptotic activity (Lizcano et al., 2000; Virdee et al., 2000), it is possible that D,L-methadone-induced downregulation of PKA activity due to ↓[cAMP]_i_ may cause reduced phosphorylation of (i) PLCβ3 at Ser1105, stimulating PLCβ3 activity and IP3R-mediated ER Ca^2+^ release, and (ii) BAD at Ser118, promoting the formation of the BCL-2-BAD complex, which neutralizes the Bcl-2 anti-apoptotic activity, allowing BAK and BAX to form pores in the outer mitochondrial membrane, inducing cyt C release, caspase activation, and apoptosis (Gavathiotis et al., 2008; Westphal et al., 2011). If this holds true, PKA may serve as a link between the two D,L-methadone-induced OPRM1-mediated apoptotic pathways: (i) G_αi_-AC-↓[cAMP]_i_-caspase pathway (Friesen et al., 2008; Friesen et al., 2013) and (ii) ↑[Ca^2+^]_i_-calpain-1-Bid-cyt C-caspase-3/12 pathway (Lee et al., 2021).

In this study, we examined the possibility that PKA is a link between the two D,L-methadone-induced apoptotic pathways in leukemic cells. Using SEM and POETIC2 ALL cells, we demonstrate that D,L-methadone-induced ER Ca^2+^ release occurs through G_αi_-dependent downregulation of AC and cAMP. This coincides with a rapid and transient downregulation of PKA-mediated inhibitory phosphorylation of PLCβ3, resulting in PLCβ3 stimulation and subsequent ER Ca^2+^ release as well as downregulation of PKA-mediated BAD Ser118 phosphorylation, which ultimately results in caspase activation and apoptosis. Our finding that PKA inhibition alone elicited ER Ca^2+^ release while PKA inhibition together with D,L-methadone treatment does not enhance ER Ca^2+^ release indicates that PKA, indeed, connects the two proposed D,L-methadone-induced apoptotic pathways in leukemic cells.

## Materials and methods

### Materials

RPMI 1640 (11875093), Opti-MEM reduced serum (31985062), MEMα (12561056), penicillin-streptomycin (15140122), phosphorylated Ser118 BAD (PA5-12550) and Mag-Fluo-4 a.m. (M14206) were from ThermoFisher Scientific (Burlington, ON, Canada). FBS (89510-186) was from VWR. The preservative-free methadone hydrochloride (NDC 17478-380-20) was from Market Drugs Medical Ltd. (Edmonton, AB). The 2,5-Di- tert-butylhydroquinone (TBHQ, 112976), 14–22 amide (myr) (476485), pertussis toxin (PTx; P7208), and Ponceau S stain (p-3504) were from Sigma-Aldrich (Oakville, ON, Canada). The 8-CPT-cAMP (ab120424) and HRP-conjugated goat antirabbit IgG (ab288151) secondary antibody were from Abcam (Toronto, ON, Canada). Forskolin (66575-29-9) was from Alomone Labs (Jerusalem, Israel). Gallein (sc-202631) and antibodies for total PLCβ3 (D-7), total BAD (C-7), and GAPDH (0411) were from Santa Cruz Biotechnology (Dallas, TX, United States of America). The antibody against phosphorylated Ser1105 PLCβ3 (2484) and HRP-conjugated goat antimouse IgG (7076) secondary antibody were from Cell Signalling (Whitby, ON, Canada).

### Cell culture

SEM and POETIC2 cell lines originated from a female 5-year-old patient diagnosed with B-cell ALL (Lee et al., 2019) and a 14-year-old patient with pre-B ALL (Lee et al., 2021), respectively, were gifts from Dr. Aru Narendran, University of Calgary. RPMI1640 and Opti-MEM reduced serum containing 10% fetal bovine serum, and 100 μg/mL penicillin/streptomycin were used to culture the SEM and POETIC2 cells, respectively, at 37°C and CO_2_ level of 5%. SEM cells stably infected with a retrovirus carrying an empty pRS vector or pRS-sh*HAP1* were generated as described previously (Lee et al., 2019). SK-N-MC neuroblastoma cells, obtained from an Askin’s tumor in a 14-year-old female patient, were cultured in MEMα containing 10% fetal bovine serum, and 100 μg/mL penicillin/streptomycin at 37°C and CO_2_ level of 5%.

### Measurement of endoplasmic reticulum (ER) Ca^2+^ release

SEM, POETIC2 and SK-N-MC cells (∼0.5 × 10^6^) grown on 0.2 mg/mL poly-L-ornithine-coated 12 mm glass coverslips were loaded with 2 μM Mag-Fluo-4 a.m. in RPMI media for 45 min. Coverslips were then transferred to a 3.5 cm glass bottom plate containing 1 mL of Ca^2+^-free Krebs-Ringer-Henseleit (KRH) buffer (25 mM HEPES, pH 7.4, 125 mM NaCl, 5 mM KCl, 6 mM glucose, 1.2 mM MgCl_2,_ and 2 μM EGTA). Ca^2+^ transients were traced using the DMi8-Film microscope at a magnification of ×20 and the LASX imaging software (Leica Microsystems). The HyD laser for confocal imaging (Leica Microsystems) was used at λ_Ex_ = 495_nm_ and λ_Em_ = 530_nm_. After obtaining stable baseline ER Ca^2+^ levels, cells were pre-treated (or not pre-treated) with 0.1 μg/mL pertussis toxin (PTx), 2 μM galleon, 2 μM 14–22 amide (myr), 1 μM 8-CPT-cAMP, or 4 μM forskolin for the indicated period of time, then treated with 2 μg/mL of D,L-methadone or 10 μM TBHQ. The average Ca^2+^ tracings were measured per sec in 10–12 individual cells following the treatment. The resulting ER Ca^2+^ release was assessed through changes in ER fluorescence: the signal-to-baseline ratio (SBR), which is simply the F/F_0_ ratio, where F = fluorescence value after stimulation and F_0_ = basal or initial fluorescence).

### Western blot analysis

Lysates of cells (1 × 10^6^) treated with 2 μg/mL D,L-methadone or 4 µM forskolin followed by 2 μg/mL D,L-methadone at the indicated times were resolved by 10% SDS-PAGE and transferred to nitrocellulose membranes (Pall Laboratory, ON), which were then immunostained for pSer1105-PLCβ3 (2484, Cell Signalling), total PLCβ3 (D-7, Santa Cruz Biotechnology), pSer118-BAD(PA5-12550, ThermoFisher Scientific), and total BAD (C-7, Santa Cruz Biotechnology). Membranes were incubated with the indicated antibody (diluted to 1:1,000) overnight at 4°C. After washing three times in tris-buffered saline containing 0.1% triton X-100 (TBS-T), membranes were incubated with an HRP-conjugated secondary antibody [1:10,000; goat antirabbit IgG (Abcam, ab288151) or goat antimouse IgG (Cell signaling, 7076)] in TBS-T for 1 h. Immunoreactive bands were detected by enhanced chemiluminescence and visualized using the Bio-Rad ChemiDoc Imager at the optimal exposure set up. Ratios of pSer1105-PLCβ3 vs. total-PLCβ3 was determined using the NIH ImageJ 1.61 software.

### Apoptotic analysis

SEM cells (^#^; 1 × 10^4^) stably infected with retrovirus carrying either pRS empty vector (^#^+pRS) or pRS-sh*HAP1* (^#^+pRS-sh*HAP1*) were seeded in 96-well plates coated with 0.02% poly-L-ornithine. Cells pre-treated with 2 μg/mL D,L-methadone or 2 μM 14–22 amide (myr), for 8 h were stained with 0.5 μg/mL annexin V-FITC (Invitrogen) and visualized at λ_ex_ = 485 nm and λ_em_ = ×530 nm and ×10 magnification using an IX71 Olympus inverted microscope (Tokyo, Japan). The percentage of apoptotic cells was calculated based on a total of 101–298 cells counted per treatment. Analysis was performed using ImageJ 1.4.1 (NIH, United States). For flow cytometry, cells (0.5 × 10^6^) pre-treated with 2 μM 14–22 amide (myr) then treated with 2 μg/mL D,L-methadone for 12 h were harvested, washed twice with 1× PBS, stained with Annexin V-FITC (2 μL) and propidium iodide (2 μL), and analysed using an Attune NxT flow cytometer (ThermoFisher Scientific, United States).

### Statistical analysis

The student’s unpaired, two-tailed *t*-test was performed at *p* < 0.05. For experiments that exceeded more than two groups or treatments, one-way Analysis of Variance (ANOVA) with Tukey Honestly Significantly Different (HSD) *post hoc* tests were conducted to uncover the statistical differences between groups or treatments.

## Results

### D,L-methadone-induced ER Ca^2+^ release in leukemic cells is dependent on G_αi_ but not G_βγ_

**FIGURE 1:**
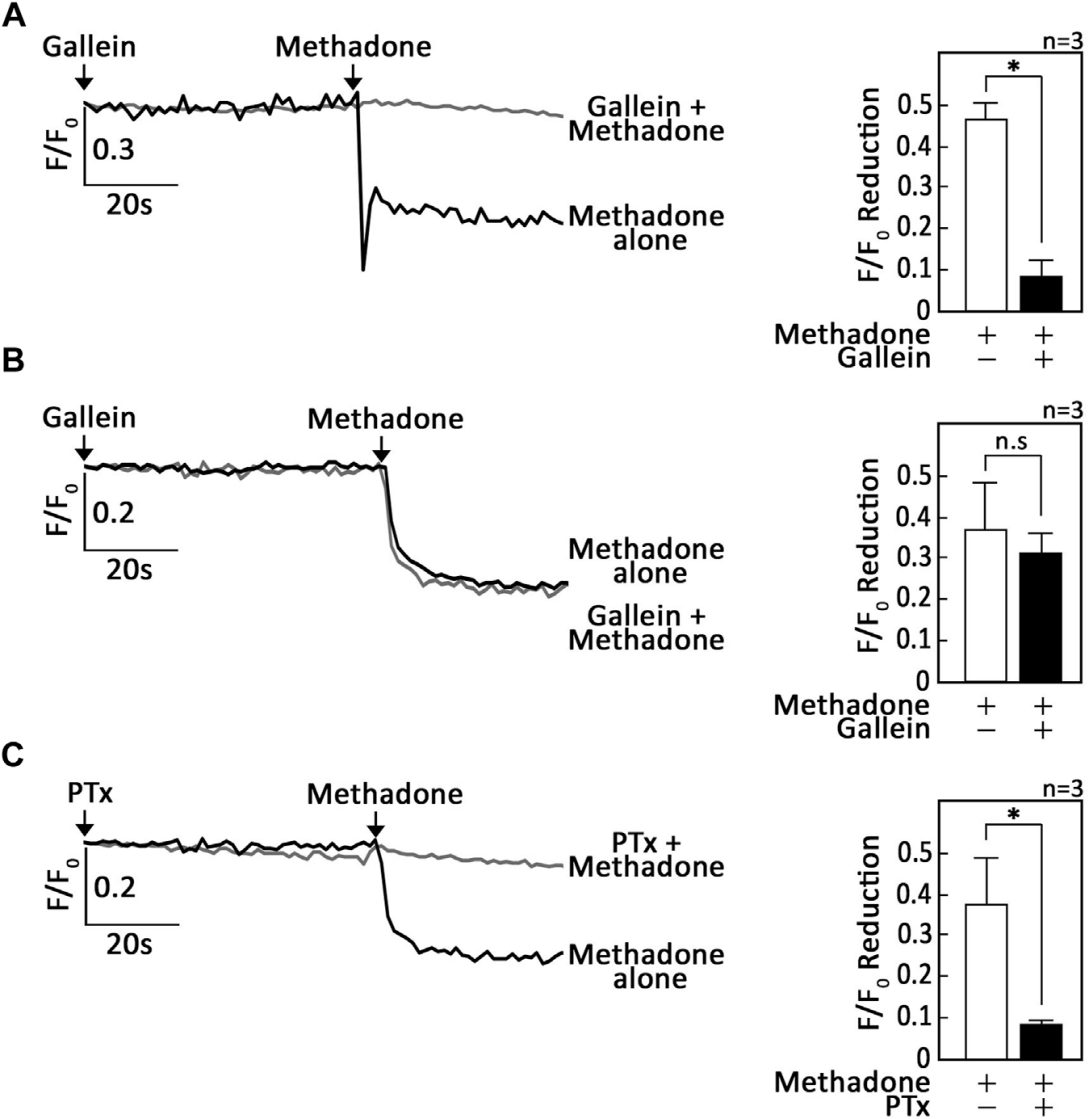
D,L-methadone-induced ER Ca^2+^ release in leukemic cells is blocked by inhibition of G_αi_, but not G_βγ_. SK-N-MC neuroblastoma **(A)** and SEM leukemic **(B,C)** cells loaded with Mag-Fluo-4 a.m. were subjected to Ca^2+^ tracings by single-cell Ca^2+^ imaging. After obtaining stable baseline ER Ca^2+^ levels, cells were pre-treated (or not pre-treated) with gallein **(A,B)** or PTx **(C)** for 60 s, then treated with D,L-methadone for 60 s to analyze ER Ca^2+^ release. The left panels show the average Ca^2+^ tracing measured per sec in 10 individual cells after gallein **(A,B)** or PTx **(C)** treatment. Data are from one of three independent experiments (n = 3) showing similar results. The charts on the right show the difference in ER Ca^2+^ release following treatment with D,L-methadone pretreated (or not pretreated) with gallein **(A,B)** or PTx **(C)**. F/F_0_ value of 30 s after D,L-methadone addition (left panel) was used to determine F/F_0_ reduction. Values are means ± SEM of the three independent experiments. *p < 0.025. N.S., not significant.

Since activation of opioid receptors has been shown to cause G_βγ_-mediated ER Ca^2+^ release via PLC in neuroblastoma (Yoon et al., 1999) and leukocytes (Celik et al., 2016; Machelska and Celik, 2018), we tested the potential involvement of G_βγ_ in D,L-methadone-induced ER Ca^2+^ release in leukemic cells. To do so, SEM (Lee et al., 2019) and POETIC2 (Lee et al., 2021) leukemic cells loaded with an ER Ca^2+^ probe (Rossi and Taylor, 2020), Mag-Fluo-4 a.m., were pre-treated with gallein, a potent G_βγ_ inhibitor (Sanz et al., 2017), then treated with D,L-methadone, and analysed for ER Ca^2+^ release. Similar analysis was performed in SK-N-MC neuroblastoma cells, which served as positive control. While gallein inhibited D,L-methadone-induced ER Ca^2+^ release in SK-N-MC neuroblastoma cells (Figure 1A), it did not do so in SEM (Figure 1B) and POETIC2 (Supplementary Figure S1B) leukemic cells, indicating that D,L-methadone-induced ER Ca^2+^ release in leukemic cells is independent of G_βγ._ Previous studies have shown that stimulation of opioid receptors by D,L-methadone activates G_αi_, which blocks adenylyl cyclase (AC) activity that in turn reduces [cAMP]_i_ (Friesen et al., 2013; Kang et al., 2017). However, it is not known whether this pathway is involved in D,L-methadone-induced ER Ca^2+^ release. Therefore, we initially tested the involvement of G_αi_ in D,L-methadone-induced ER Ca^2+^ release. Treatment with PTx, a potent G_αi_ inhibitor (Wu and Wong, 2005), completely abolished D,L-methadone-induced ER Ca^2+^ release (Figure 1C; Supplementary Figure S1C), indicating that this process involves G_αi_.

### D,L-methadone-induced ER Ca^2+^ release in leukemic cells results from downregulation of AC, cAMP and PKA

**FIGURE 2:**
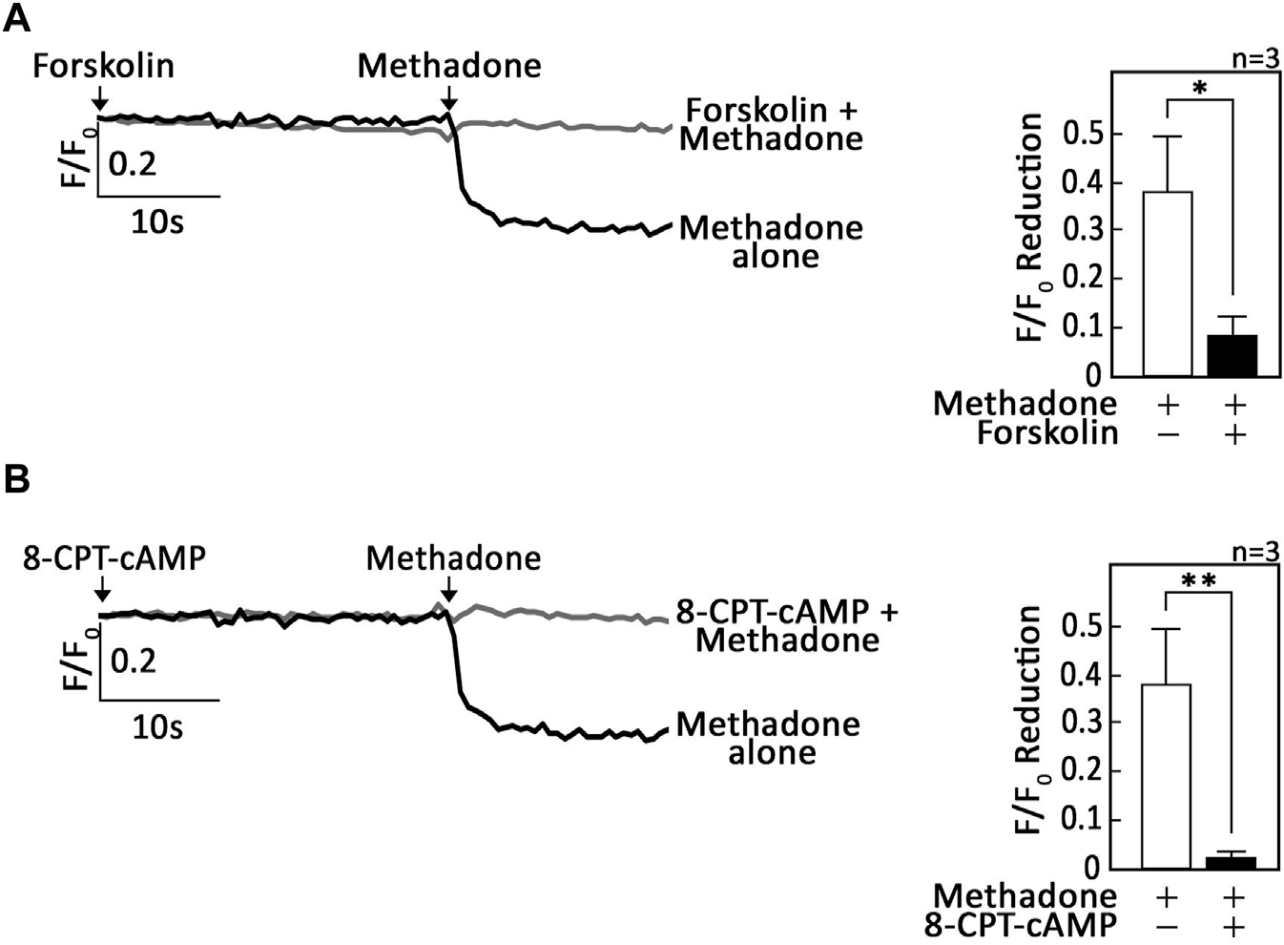
Stimulation of AC with forskolin or treatment with exogenous 8-CPT-cAMP inhibits D,L-methadone-induced ER Ca^2+^ release. SEM leukemic cells loaded with Mag-Fluo-4 a.m. were subjected to Ca^2+^ tracings by single-cell Ca^2+^ imaging. After obtaining stable baseline ER Ca^2+^ levels, cells were pre-treated (or not pre-treated) with forskolin **(A)** or 8-CPT-cAMP **(B)** for 30 s then treated with D,L-methadone for 30 s, to analyze ER Ca^2+^ release. The left panels show the average Ca^2+^ tracing measured per sec in 10 individual cells after forskolin **(A)** or 8-CPT-cAMP **(B)** treatment. Data are from one of three independent experiments (n = 3) showing similar results. The charts on the right show the difference in ER Ca^2+^ release following treatment with D,L-methadone pretreated (or not pretreated) with forskolin **(A)** or 8-CPT-cAMP **(B)**. F/F_0_ value of 15 s after D,L-methadone addition (left panel) was used to determine F/F_0_ reduction. Values are means ± SEM of the three independent experiments. *p < 0.025. **p < 0.05.

We then tested whether the G_αi_-dependent D,L-methadone-induced ER Ca^2+^ release involves AC and cAMP. To do so, SEM and POETIC2 leukemic cells loaded with Mag-Fluo-4 a.m. were pre-treated with forskolin, an AC activator, or 8-CPT-cAMP, an exogenous cAMP analogue. Cells were then treated with D,L-methadone and analysed for ER Ca^2+^ release. As shown in Figures 2A, B (SEM cells) as well as Supplementary Figures S2A, S2B (POETIC2 cells), forskolin and 8-CPT-cAMP, respectively, inhibited D,L-methadone-induced ER Ca^2+^ release, indicating AC and cAMP involvement in this process.

**FIGURE 3:**
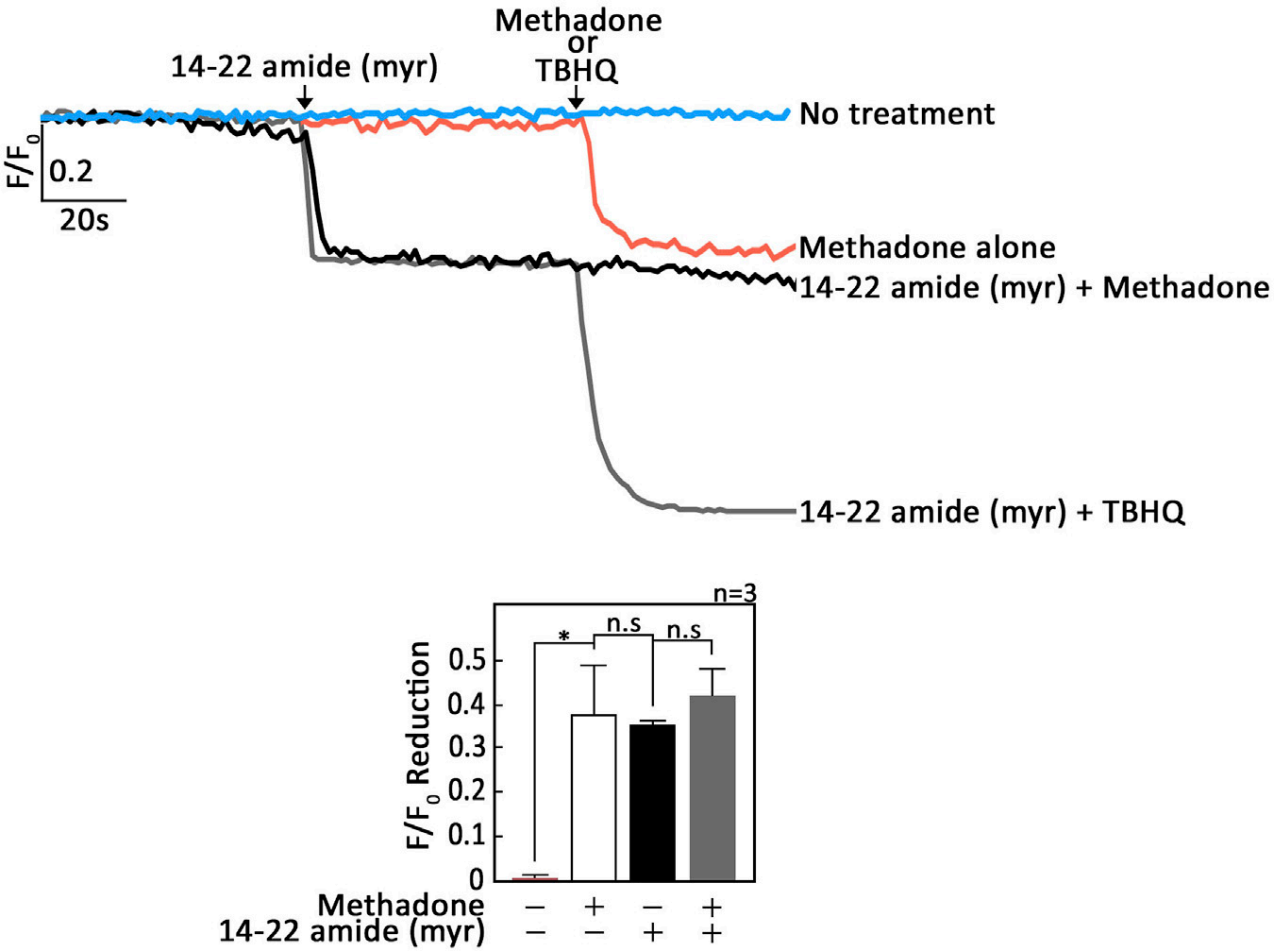
Treatment with 14–22 amide (myr) alone elicits ER Ca^2+^ release in leukemic cells and subsequent treatment with D,L-methadone does not cause additional ER Ca^2+^ release. SEM leukemic cells loaded with Mag-Fluo-4 a.m. were subjected to Ca^2+^ tracing by single-cell Ca^2+^ imaging. After obtaining stable baseline ER Ca^2+^ levels, cells were pretreated (or not pretreated) with 14–22 amide (myr) for 60 s then treated with D,L-methadone or TBHQ for 60 s, to analyze ER Ca^2+^ release. The left panels show the average Ca^2+^ tracing measured per sec in 10 individual cells after 14–22 amide (myr) treatment. Treatment with TBHQ, an ER Ca^2+^ pump inhibitor, caused further ER Ca^2+^ release, indicating that these cells were viable during analysis. Data are from one of three independent experiments (n = 3) showing similar results. The chart on the bottom shows the difference in ER Ca^2+^ release following treatment with D,L-methadone pretreated (or not pretreated) with 14–22 amide (myr). F/F_0_ value of 30 s after D,L-methadone and/or 14–22 amide (myr) addition (left panel) was used to determine F/F_0_ reduction. Values are means ± SEM of the three independent experiments. *p < 0.025. N.S., not significant.

Since cAMP signaling regulates [Ca^2+^]_i_ through inhibitory phosphorylation of PLCβ3 by PKA (Liu and Simon, 1996; Ali et al., 1998; Yue et al., 1998), it is possible that OPRM1 stimulation by D,L-methadone also involves PKA inhibition. If so, we expect that treatment with 14–22 amide (myr), a myristylated cell permeable PKA-specific inhibitor (Dalton et al., 2005), alone will evoke ER Ca^2+^ release. In addition, pre-treatment with 14–22 amide (myr) will not cause further increase in D,L-methadone-induced ER Ca^2+^ release. Indeed, as shown in Figure 3; Supplementary Figure S3 14-22 amide (myr) alone triggered ER Ca^2+^ release and subsequent treatment with D,L-methadone did not cause additional ER Ca^2+^ release. These findings indicate that G_αi_-AC-cAMP-dependent D,L-methadone-induced ER Ca^2+^ release involves PKA inhibition, which can circumvent the upstream D,L-methadone-OPRM1-AC-cAMP step. Treatment with TBHQ, an ER Ca^2+^ pump inhibitor, caused further ER Ca^2+^ release, indicating that these cells were viable during analysis (Figure 3; Supplementary Figure S3).

### D,L-methadone-induced ER Ca^2+^ release in leukemic cells is associated with downregulation of PKA-mediated (i) PLCβ3 inhibitory phosphorylation at Ser1105 and (ii) BAD phosphorylation at Ser118

**FIGURE 4:**
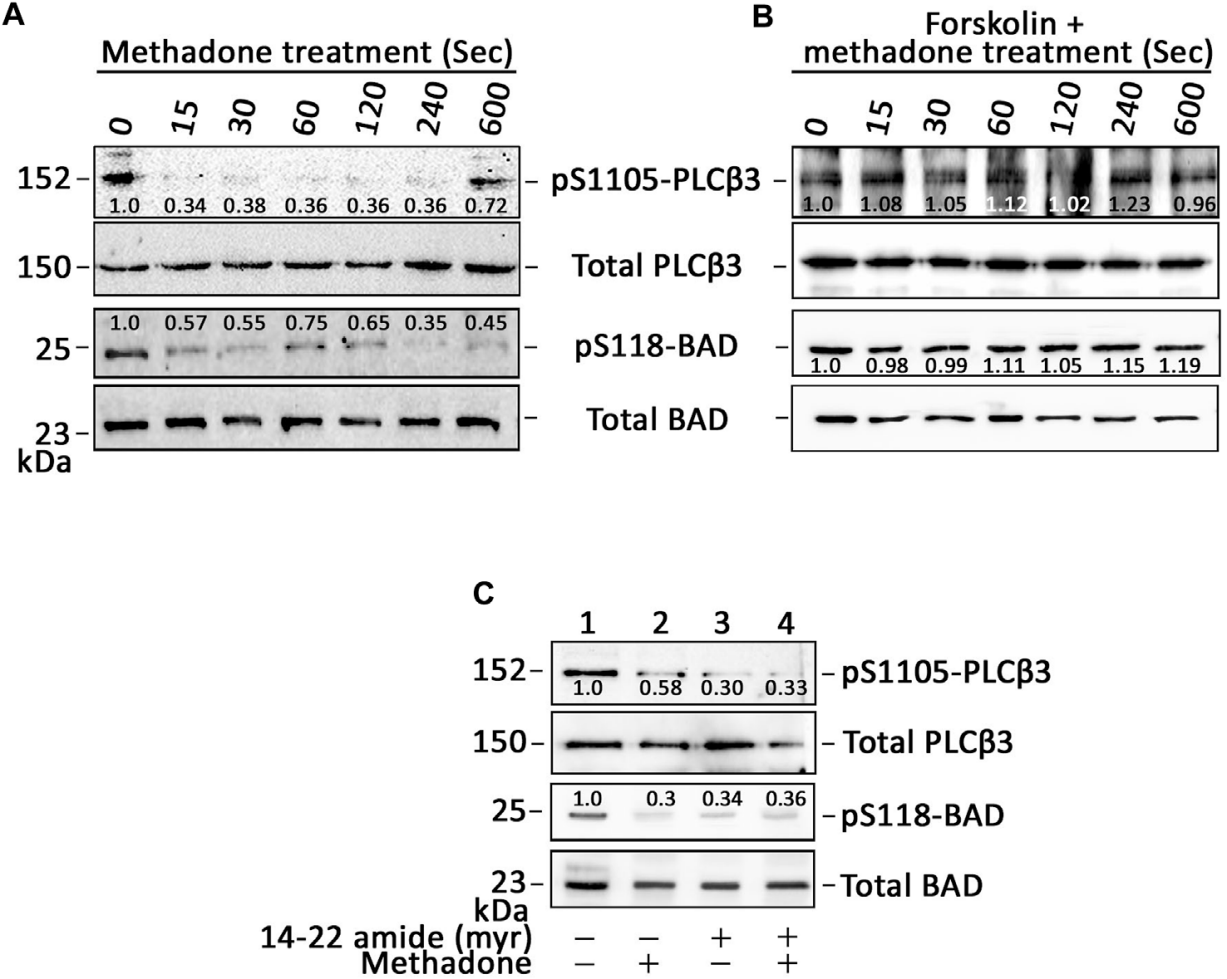
D,L-methadone-induced ER Ca^2+^ release in leukemic cells is associated with downregulation of PLCβ3 Ser1105 inhibitory phosphorylation and BAD phosphorylation at Ser118. **(A–C)** Lysates of SEM cells pre-treated (or not pre-treated; A) with forskolin **(B)** or 14–22 amide (myr) **(C)** then treated with D,L-methadone were subjected to SDS-PAGE and immunoblotting for pSer1105-PLCβ3 and total PLCβ3, and/or pSer118-BAD and total BAD. In C, lysates of cells treated with D,L-methadone for 120 s were used. The numbers under the pSer1105-PLCβ3 and pSer118-BAD bands represent the relative intensity ratios of pSer1105-PLCβ3 or pSer118-BAD vs. total PLCβ3 or BAD bands, respectively, with values at time 0 normalized to 1. Representative blots are from one of three independent experiments (n = 3) showing similar results.

Since stimulation of opioid receptors causes ER Ca^2+^ release via PLC (Yoon et al., 1999; Machelska and Celik, 2018), we examined whether D,L-methadone downregulates PKA-mediated inhibitory phosphorylation of PLCβ3 at Ser1105, which is necessary for inducing PLC-IP3/IP3R-mediated ER Ca^2+^ release (Zhong et al., 2008). To do so, lysates of cells treated with D,L-methadone were subjected to SDS-PAGE and immunoblotting for pSer1105-PLCβ3. As shown in Figure 4A; Supplementary Figures S4A, S6A, D,L-methadone drastically reduced the level of pSer1105-PLCβ3 after 15 s of treatment, which coincides with the prompt ER Ca^2+^ release upon D,L-methadone treatment as shown in Figures 1B, C; Supplementary Figure S1. As expected, pre-treatment with forskolin did not affect D,L-methadone-induced PLCβ3 phosphorylation at Ser1105 (Figure 4B; Supplementary Figures S4A, S6B), which is consistent with the loss of D,L-methadone-induced ER Ca^2+^ release upon forskolin treament (Figure 2A; Supplementary Figure S2A). Since cAMP activates protein kinase A (PKA), which phosphorylates BAD at Ser118, disrupting BCL-2-BAD interaction, which stimulates the BCL-2 anti-apoptotic activity (Lizcano et al., 2000; Virdee et al., 2000), we sought to examine whether D,L-methadone also downregulates BAD phosphorylation at Ser118. Figure 4A; Supplementary Figures S4, S6A shows that as with pSer1105-PLCβ3, the level of pSer118-BAD was reduced immediately upon D,L-methadone treatment. Pre-treatment with forskolin did not affect D,L-methadone-induced BAD phosphorylation at Ser118 (Figure 4B; Supplementary Figures S4, S6B). To determine the significance of PKA inhibition in PLCβ3 activation, lysates of cells pre-treated with 14–22 amide (myr) then treated with D,L-methadone were subjected to SDS-PAGE and immunoblotting for pSer1105-PLCβ3 and pSer118-BAD. As shown in Figure 4C; Supplementary Figures S5, S6C, treatment with D,L-methadone or 14–22 amide (myr) considerably reduced the phosphorylation of PLCβ3 at Ser1105 and BAD at Ser118, indicating that such phosphorylation occurs via PKA. Our observation that 14–22 amide (myr) alone caused considerable decrease in PLCβ3 phosphorylation at Ser1105 and subsequent treatment with D,L-methadone did not cause further decrease in PLCβ3 phosphorylation at Ser1105 is consistent with our finding that 14–22 amide (myr) alone triggered ER Ca^2+^ release, and suggests that D,L-methadone-induced OPRM1-mediated ER Ca^2+^ release occurs via the G_αi_-AC-cAMP-PKA-PLC pathway.

### PKA inhibition alone triggers leukemic cell apoptosis

**FIGURE 5:**
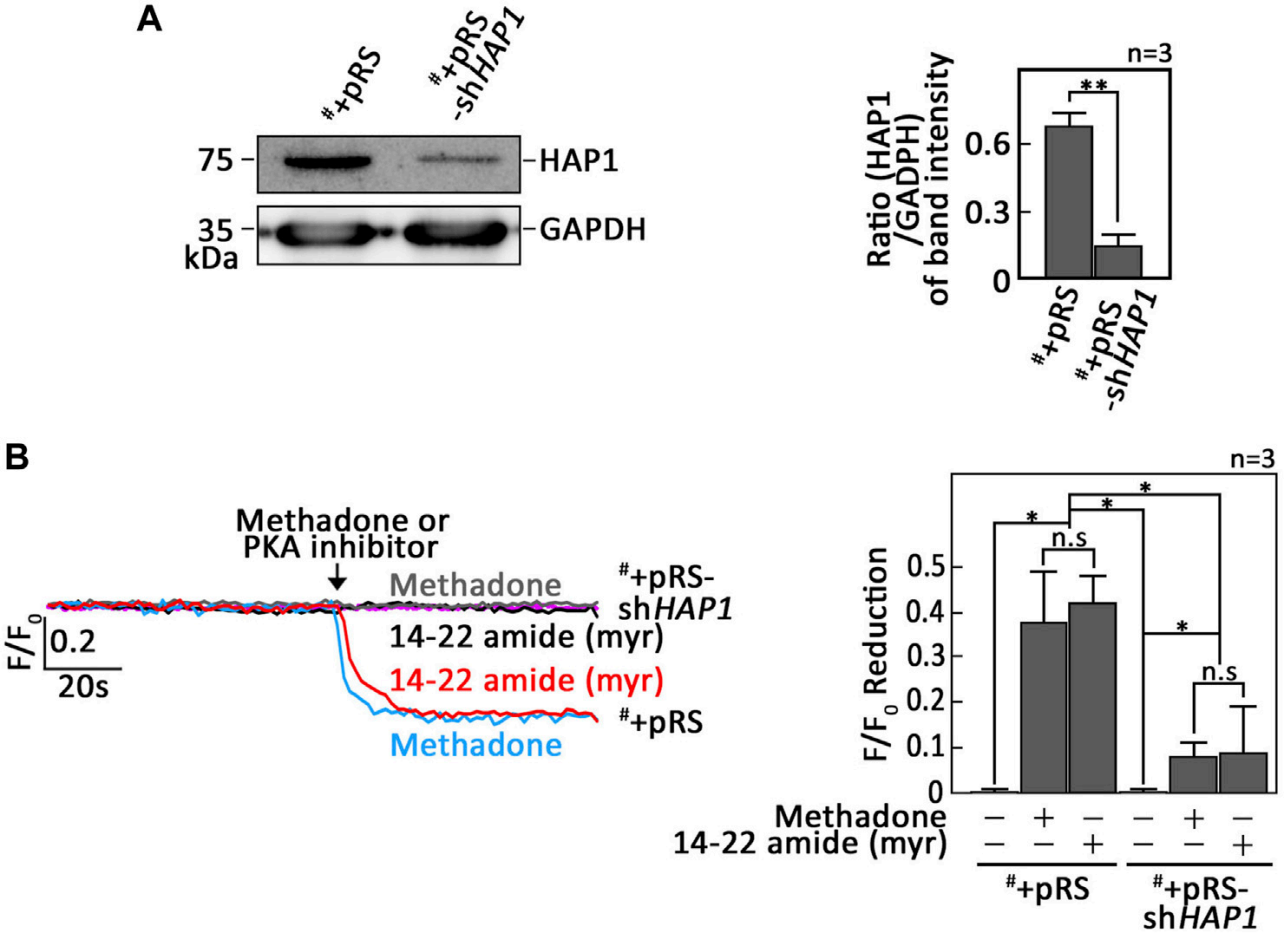
Leukemic cells stably depleted of HAP1 inhibit ER Ca^2+^ release upon stimulation with D,L-methadone, or treatment with 14–22 amide (myr). **(A)** Lysates of SEM cells (^#^) infected with retrovirus carrying pRS empty vector (^#^+pRS) or pRS-sh*HAP1* (^#^+pRS-sh*HAP1*) were resolved by SDS-PAGE and immunoblotted for HAP1. GAPDH blot was used as loading control. The right panel shows the ratios of HAP1 vs. GAPDH levels, which were measured by densitometric analysis of the blots using NIH ImageJ 1.61. GAPDH levels were normalized to 1.0. Standard deviation of the HAP1 vs. GAPDH ratio was calculated from the three sets of experiments. **(B)** ^#^+pRS and ^#^+pRS-sh*HAP1* cells loaded with Mag-Fluo-4 a.m. were subjected to Ca^2+^ tracing by single-cell Ca^2+^ imaging. After obtaining stable baseline ER Ca^2+^ levels, cells were (or not treated: purple colored trace) treated with 14–22 amide (myr) or D,L-methadone for 60 s, to analyze ER Ca^2+^ release. The left panel shows the average Ca^2+^ tracing per sec from 10 individual cells before and after treatment. Data are from one of three independent experiments (n = 3) showing similar results. The chart on the right shows the difference in ER Ca^2+^ release in ^#^+pRS and ^#^+pRS-sh*HAP1* cells following treatment with 14–22 amide (myr) or D,L-methadone. F/F_0_ value of 30 s after D,L-methadone or 14–22 amide (myr) addition (left panel) was used to determine F/F_0_ reduction. Values are means ± SEM of the three independent experiments. *p < 0.025. **p < 0.05. N.S., not significant.

**FIGURE 6:**
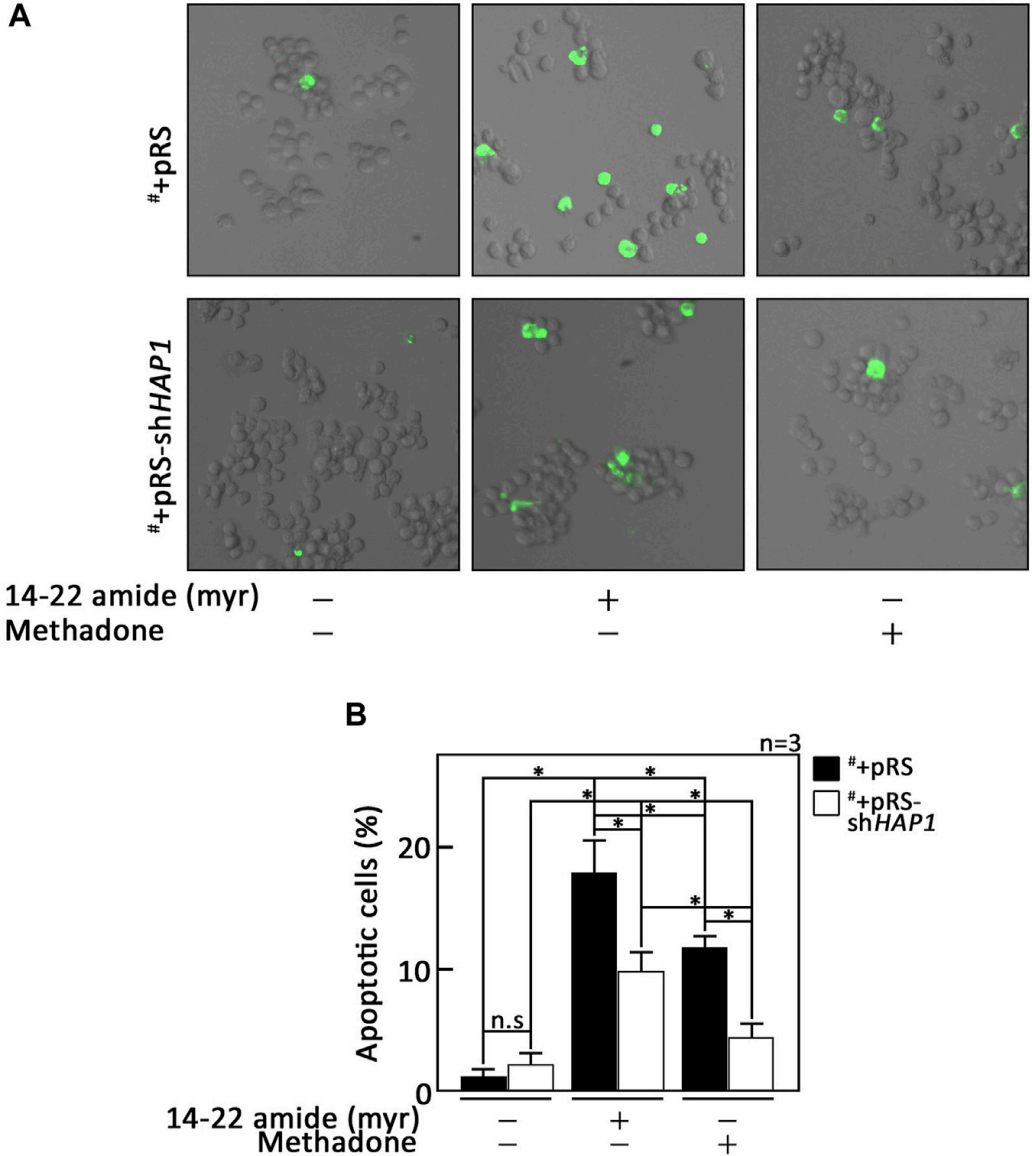
PKA inhibition alone causes leukemic cell apoptosis. **(A)** ^#^+pRS and ^#^+pRS-sh*HAP1* cells treated with 14–22 amide (myr) or D,L-methadone for 8 h were stained with FITC-annexin V and subjected to fluorescence microscopy. **(B)** The percentage of apoptotic cells was calculated based on a total of 101–298 cells counted per treatment. Values are means ± SEM from three independent experiments (n = 3) that showed similar results. *p < 0.025. N.S., not significant.

As our data indicated that PKA inhibition is a central step in D,L-methadone-induced apoptosis that involves PLCβ3 activation and BAD Ser118 phosphorylation, we sought to examine whether treatment with 14–22 amide (myr) alone causes leukemic cell apoptosis. To do so, cells treated with 14–22 amide(myr) for 8 h were stained with FITC-annexin V and subjected to fluorescence microscopy. The percentage of apoptotic cells was calculated. For negative control, we used SEM cells (^#^) stably depleted of HAP1 by infection with retrovirus carrying pRS-sh*HAP1* (^#^+pRS-sh*HAP1*). Lack of HAP1 (Figure 5A), a key component of the functional IP3R/HAP1/Htt ternary complex (Tang et al., 2003; Lee et al., 2019), inhibits ER Ca^2+^ release upon stimulation with D,L-methadone or treatment with 14–22 amide (myr) (Figure 5B). In contrast, D,L-methadone and 14–22 amide (myr) cause a similar extent of ER Ca^2+^ release in ^#^+pRS cells (Figure 5B). As shown in Figure 6, ^#^+pRS cells show greater apoptosis compared to ^#^+pRS-sh*HAP1* cells upon treatment with 14–22 amide (myr) (17.7% vs. 9.8%) or D,L-methadone (11.6% vs. 4.5%). It is interesting that 14–22 amide (myr) causes greater apoptosis compared to D,L-methadone in both ^#^+pRS and ^#^+pRS-sh*HAP1* cells (17.7% vs. 11.6% and 9.8% vs. 4.5%, respectively). Similar results were also obtained in POETIC2 cells (Supplementary Figure S7). Together, these findings indicate that PKA inhibition by 14–22 amide (myr) effectively kills ALL cells.

## Discussion

**FIGURE 7:**
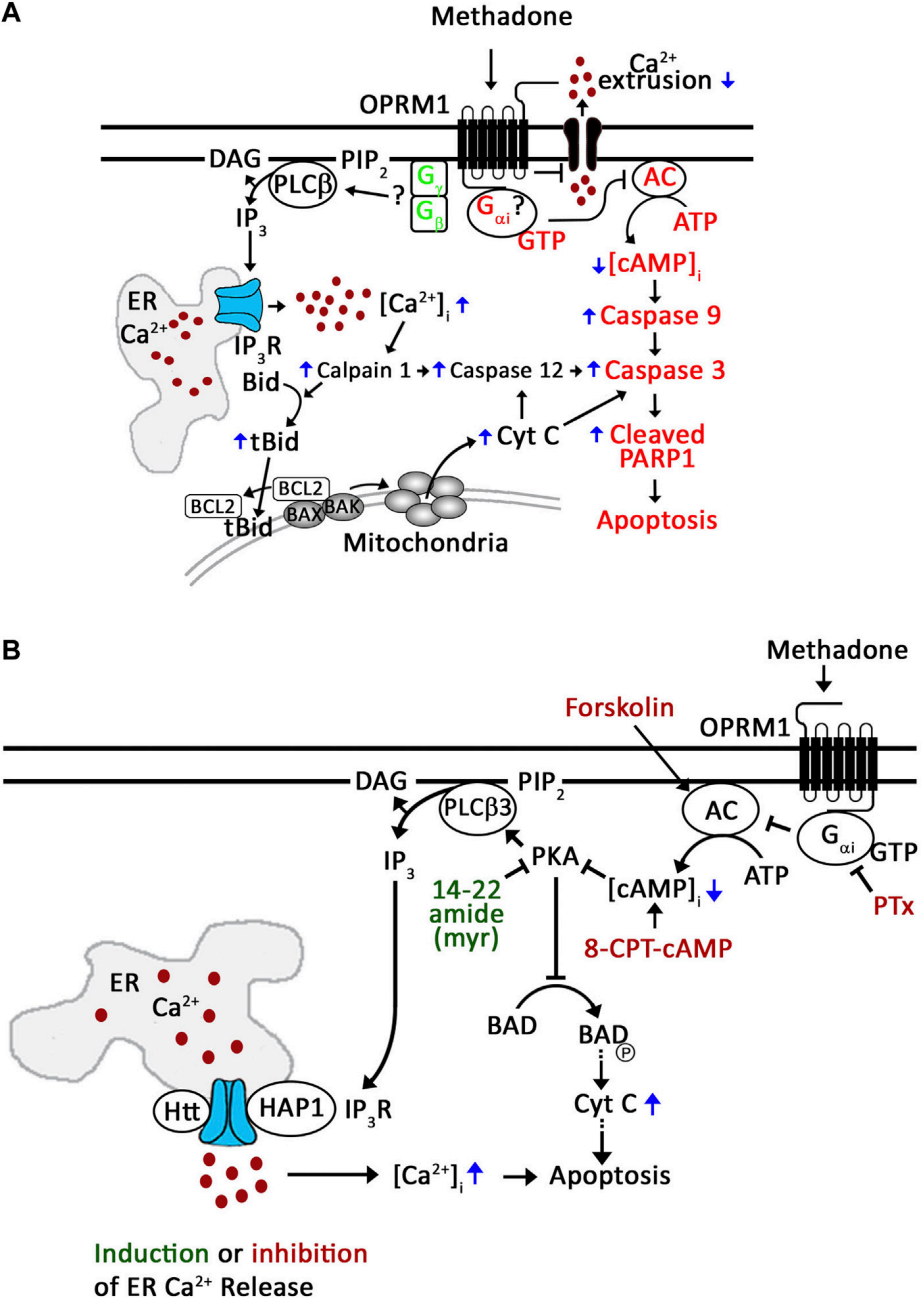
Proposed mechanism for D,L-methadone-induced OPRM1-mediated apoptosis in leukemic cells. **(A)** Friesen et al. (2013) has shown that D,L-methadone induces leukemic cell apoptosis through the G_αi_-AC-↓[cAMP]_i_-caspase pathway (in red). Our previous studies showed that D,L-methadone specifically stimulates the opioid receptor mu1 subtype, OPRM1, in leukemic cells (Lee et al., 2021); D,L-methadone activation of OPRM1 causes increased [Ca^2+^]_i_ by enhancing IP3R-mediated ER Ca^2+^ release and decreasing Ca^2+^ efflux, upregulating the Ca^2+^-mediated calpain-1-Bid-cyt C-caspase-3/12 apoptotic pathway (in black) (Lee et al., 2021). Together, these findings point to two D,L-methadone-induced OPRM-mediated apoptotic pathways: (i) G_αi_-AC-↓[cAMP]_i_-caspase pathway (Friesen et al., 2013) and (ii) ↑[Ca^2+^]_i_-calpain-1-Bid-cyt C-caspase-3/12 pathway (Lee et al., 2021). Since activation of opioid receptors causes G_βγ_-mediated ER Ca^2+^ release via PLC in neuroblastoma cells (Yoon et al., 1999) and leukocytes (Celik et al., 2016; Machelska and Celik, 2018), in the latter pathway, we proposed the involvement of G_βγ_ (in green) in D,L-methadone-induced ER Ca^2+^ release in leukemic cells (Lee et al., 2021). **(B)** In the current study, we demonstrate that D,L-methadone-induced ER Ca^2+^ release in ALL cells results from G_αi_-dependent downregulation of AC and cAMP. PKA inhibition elicits ER Ca^2+^ release and overrides D,L-methadone-induced ER Ca^2+^ release, indicating that PKA inhibition is a central step in D,L-methadone-induced ER Ca^2+^ release. This is consistent with decrease in PKA-dependent (i) inhibitory phosphorylation of PLCβ3 at Ser1105 that leads to PLCβ3 activation and ER Ca^2+^ release, and (ii) BAD Ser118 phosphorylation, which ultimately result in caspase activation and apoptosis. Thus, D,L-methadone-induced ER Ca^2+^ release and subsequent apoptosis in ALL cells is mediated by G_αi_-dependent downregulation of the AC-cAMP-PKA-PLCβ3/BAD pathway. Small molecular activators/inhibitors shown in red inhibit ER Ca^2+^ release; 14–22 amide (myr) shown in green induces ER Ca^2+^ release.

In recent years, the molecular mechanism by which D,L-methadone induces leukemic cell apoptosis has been investigated (Friesen et al., 2013; Lee et al., 2021). Friesen et al. (2013) have shown that D,L-methadone, which stimulates opioid receptors, causes ALL cell apoptosis through G_αi_-AC, decrease in [cAMP]_i_ and subsequent activation of caspase-9 and -3 (Figure 7A, in red). However, the identity of the specific opioid receptor(s) targeted by D,L-methadone was not defined. Through unbiased genome-wide RNAi screening and knockdown studies, we previously found that presence or absence of the μ1 opioid receptor subtype, OPRM1, determines the fate of ALL cells following L-asparaginase treatment, i.e., presence leads to apoptosis while absence leads to survival or resistance (Kang et al., 2017). In further studies, we found that OPRM1 is targeted by D,L-methadone to induce leukemic cell apoptosis (Lee et al., 2021). In fact, OPRM1 loss inhibits D,L-methadone-induced ALL cell apoptosis (Lee et al., 2021). As shown in Figures 7A, D,L-methadone activation of OPRM1 causes a lethal rise in [Ca^2+^]_i_ by stimulating IP3R-induced ER Ca^2+^ release through PLCβ and decreasing Ca^2+^ efflux, leading to upregulation of the Ca^2+^-mediated calpain-1-Bid-cyt C-caspase-3/12 apoptotic pathway (Figure 7A, in black) (Lee et al., 2021). Based on the model that D,L-methadone causes G_βγ_-mediated [Ca^2+^]_i_ increase in leukemic cells (Friesen et al., 2013), and the fact that in leukocytes (Celik et al., 2016; Machelska and Celik, 2018) and neuroblastoma cells (Yoon et al., 1999), opioid causes an increase in [Ca^2+^]_i_ through G_βγ_, it is possible that PLCβ-mediated ER Ca^2+^ release is triggered through G_βγ_-coupled OPRM1 (Figure 7A). Since reduced [cAMP]_i_, resulting from G_αi_-mediated inhibition of AC, causes loss of PKA activation, which (i) stimulates PLCβ3 and subsequent IP3/IP3R-mediated ER Ca^2+^ release (Yue et al., 1998) and (ii) promotes the formation of the BCL-2-BAD complex, which neutralizes the Bcl-2 anti-apoptotic activity, allowing BAK and BAX to form pores in the outer mitochondrial membrane, inducing cyt C release, caspase activation, and apoptosis (Gavathiotis et al., 2008; Westphal et al., 2011), it is also possible that PKA inhibition, not G_βγ_ activation, causes this process following stimulation with D,L-methadone (Figure 7A). The current study tested these possibilities.

Upon testing the possibility that G_βγ_-coupled OPRM1 triggers PLCβ3-mediated ER Ca^2+^ release, we found that PTx, but not gallein, inhibited D,L-methadone-induced ER Ca^2+^ release, indicating that this process is dependent on G_αi_ but not G_βγ_. This was corroborated by our data showing that stimulation of AC with forskolin (Insel and Ostrom, 2003), and treatment with exogenous 8-CPT-cAMP inhibited D,L-methadone-induced ER Ca^2+^ release, indicating that D,L-methadone caused G_αi_-mediated downregulation of AC activity, resulting in reduced [cAMP]_i_. While G_βγ_ in pancreatic acini cells was shown to also directly activate IP3R independent of IP3 production, causing ER Ca^2+^ release (Zeng et al., 2003), the inability of gallein to inhibit D,L-methadone-induced ER Ca^2+^ release, and the ability of PTx to completely inhibit D,L-methadone-induced ER Ca^2+^ release indicate that D,L-methadone-induced ER Ca^2+^ release in leukemia cells does not involve direct activation of IP3R by G_βγ_. The requirement for PKA inhibition in D,L-methadone-induced OPRM1-mediated ER Ca^2+^ release was proven by our data showing that the 14–22 amide (myr) PKA inhibitor alone evoked ER Ca^2+^ release, and that subsequent treatment with D,L-methadone did not cause additional ER Ca^2+^ release. The involvement of G_αi_-AC-cAMP-PKA-PLC was further supported by our observations that D,L-methadone triggered immediate downregulation of PKA-mediated PLCβ3 inhibitory phosphorylation at Ser1105, which is required for eliciting IP3/IP3R-mediated ER Ca^2+^ release (Zhong et al., 2008), and that pre-treatment with forskolin did not affect D,L-methadone-induced PLCβ3 phosphorylation at Ser1105. Potentially, protein phosphatase (PP)1/PP2A (Chiang et al., 2003; Zhong et al., 2008) is involved in the dephosphorylation of PLCβ3 at Ser1105 following D,L-methadone-induced inactivation of PKA. Our finding that D,L-methadone also downregulated BAD phosphorylation at Ser118 is consistent with previously published works showing cytochrome C release and caspase activation in D,L-methadone-induced ALL cell apoptosis (Lee et al., 2021). These findings indicate that PKA serves as a link between the two previously described D,L-methadone-induced OPRM-mediated apoptotic pathways: (i) G_αi_-AC-↓[cAMP]_i_-caspase (Friesen et al., 2013) and ↑[Ca^2+^]_i_-calpain-1-Bid-cyt C-caspase-3/12^16^. Our data showing that the 14–22 amide (myr) PKA inhibitor alone reduced the levels of pSer118-BAD and pSer1105-PLCβ3, which are part of the above two apoptotic pathways, respectively (Figure 7B), led us to examine whether 14–22 amide (myr) induces ALL cell apoptosis. In fact, we found that PKA inhibition using 14–22 amide (myr) effectively killed ALL cells. The lesser extent of apoptosis in cells depleted of HAP1 (^#^+pRS-sh*HAP1*) compared to control cells (^#^+pRS) upon treatment with 14–22 amide (myr) or D,L-methadone is likely due to the inability of ^#^+pRS-sh*HAP1* cells to induce IP3R-mediated ER Ca^2+^ release. Since 14–22 amide (myr) and D,L-methadone caused a similar extent of ER Ca^2+^ release in ^#^+pRS cells, our observation that 14–22 amide (myr) causes greater apoptosis compared to D,L-methadone in both ^#^+pRS and ^#^+pRS-sh*HAP1* cells may be attributed to the greater efficiency of the 14–22 amide (myr) PKA inhibitor in downregulating BAD Ser118 phosphorylation and stimulating cyt C release. While PKA and PLCβ are involved in autophagy, there is no report indicating that D,L-methadone affects autophagy in ALL cells.

While lack of OPRM1 causes ALL cell resistance to asparaginase (Kang et al., 2017), the fact that PKA inhibition can circumvent the upsteam OPRM1-AC-cAMP step suggests that PKA may be targeted for therapy in L-asparaginase-resistant ALL. In addition, although D,L-methadone holds promise as future therapy for ALL (Friesen et al., 2013; Michalska et al., 2017; Onken et al., 2017), it is clear that PKA may also be targeted for therapy in both D,L-methadone-refractory and -sensitive ALL.

## Data Availability

The raw data supporting the conclusion of this article will be made available by the authors, without undue reservation.

## Supplementary material

SUPPLEMENTARY FIGURE S1 G_αi_-dependent D,L-methadone-induced ER Ca^2+^ release in POETIC2 cells. POETIC2 cells loaded with Mag-Fluo-4 AM were subjected to Ca^2+^ tracing by single-cell Ca^2+^ imaging. After obtaining stable baseline ER Ca^2+^ levels, cells were pretreated (or not pre-treated, **(A)** with gallein **(B)** or PTx **(C)** for 2 min, then treated with D,L-methadone for 2 min to analyze ER Ca^2+^ release. The left panels show the average Ca^2+^ tracing measured per sec in 12 individual cells after gallein **(B)** or PTx **(C)** treatment. Data are from one of three independent experiments (n = 3) showing similar results. The charts on the right show the difference in ER Ca^2+^ release following treatment with D,L-methadone in cells pretreated (or not pretreated, **(A)** with gallein **(B)** or PTx **(C)**. F/F_0_ value of 40 s after D,L-methadone addition (left panel) was used to determine F/F_0_ reduction. Values are means ± SEM of the three independent experiments. **p* < 0.025. N.S., not significant.

SUPPLEMENTARY FIGURE S2 Inhibition of D,L-methadone-induced ER Ca^2+^ release by forskolin or 8-CPT-cAMP in POETIC2 cells. POETIC2 leukemic cells loaded with Mag-Fluo-4 AM were subjected to Ca^2+^ tracings by single-cell Ca^2+^ imaging. After obtaining stable baseline ER Ca^2+^ levels, cells were pre-treated (or not pre-treated) with forskolin **(A)** or 8-CPT-cAMP **(B)** for 40 s then treated with 1 µg/ml D,L-methadone for 70 s, to analyze ER Ca^2+^ release. The left panels show the average Ca^2+^ tracing measured per sec in 12 individual cells after forskolin **(A)** or 8-CPT-cAMP **(B)** treatment. Data are from one of three independent experiments (n = 3) showing similar results. The charts on the right show the difference in ER Ca^2+^ release following treatment with D,L-methadone pretreated (or not pretreated) with forskolin **(A)** or 8-CPT-cAMP **(B)**. F/F_0_ value of 30 s after D,L-methadone addition (left panel) was used to determine F/F_0_ reduction. Values are means ± SEM of the three independent experiments. **p* < 0.05.

SUPPLEMENTARY FIGURE S3 ER Ca^2+^ release by 14-22 amide (myr) alone in POETIC2 cells. POETIC2 leukemic cells loaded with Mag-Fluo-4 AM were subjected to Ca^2+^ tracing by single-cell Ca^2+^ imaging. After obtaining stable baseline ER Ca^2+^ levels, cells were pretreated (or not pretreated) with 14-22 amide (myr) for 30 s then treated with D,L-methadone or TBHQ for 100 s, to analyze ER Ca^2+^ release. The left panels show the average Ca^2+^ tracing measured per sec in 10 individual cells after 14-22 amide (myr) treatment. Treatment with TBHQ, an ER Ca^2+^ pump inhibitor, caused further ER Ca^2+^ release, indicating that these cells were viable during analysis. Data are from one of three independent experiments (n = 3) showing similar results. The chart on the bottom shows the difference in ER Ca^2+^ release following treatment with D,L-methadone pretreated (or not pretreated) with 14-22 amide (myr). F/F_0_ value of 30 s after D,L-methadone and/or 14-22 amide (myr) addition (left panel) was used to determine F/F_0_ reduction. Values are means ± SEM of the three independent experiments. **p* < 0.025. N.S., not significant.

SUPPLEMENTARY FIGURE S4 The bar graphs in **(A,B)** represent the levels of pSer1105-PLCβ3 vs total PLCβ3 ratios in Figure 4A (left upper two panels) and 4B (right upper two panels) and the levels of pSer118-BAD vs total BAD ratios in Figure 4A (left lower two panels) and 4B (right lower two panels), respectively. Values are means ± SEM of the three independent experiments (n = 3). **p* < 0.05. N.S., not significant.

SUPPLEMENTARY FIGURE S5 The bar graphs in the left and right represent the levels of pSer1105-PLCβ3 vs total PLCβ3 ratios in Figure 4C (upper two panels) and the levels of pSer118-BAD vs total BAD ratios in Figure 4C (lower two panels), respectively. Values are means ± SEM of the three independent experiments (n = 3). **p* < 0.05. N.S., not significant.

SUPPLEMENTARY FIGURE S6 Downregulation of PLCβ3 Ser1105 inhibitory phosphorylation and BAD phosphorylation at Ser118 in POETIC2 leukemic cells that induce ER Ca^2+^ release upon D,L-methadone treatment. **(A–C)** Lysates of POETIC2 cells pre-treated or not pre-treated; **(A)** with forskolin **(B)** or 14-22 amide (myr) **(C)** then treated with D,L-methadone were subjected to SDS-PAGE and immunoblotting for pSer1105-PLCβ3 and total PLCβ3, and/or pSer118-BAD and total BAD. In **(C)**, lysates of cells treated with D,L-methadone for 120 s were used. The numbers under the pSer1105-PLCβ3 and pSer118-BAD bands represent the relative intensity ratios of pSer1105-PLCβ3 or pSer118-BAD vs total PLCβ3 or BAD bands, respectively, with values at time 0 normalized to 1. Data are from one of two independent experiments (n = 2) showing similar results.

SUPPLEMENTARY FIGURE S7 PKA inhibition-induced ALL cell apoptosis analyzed by flow cytometry. ^#^+pRS and ^#^+pRS-sh*HAP1* cells pre-treated (or not pre-treated) with 14-22 amide (myr) then treated (not treated) with D,L-methadone for 12 h were stained with PI and FITC-annexin V and subjected to flow cytometry as described in Materials and Methods. Values are means ± SEM from three independent experiments (n = 3). **p* < 0.05. N.S., not significant.
