# Supplementary figures and images for: PKA inhibition is a central step in D,L-methadone-induced ER Ca2+ release and subsequent apoptosis in acute lymphoblastic leukemia
Source: Front Cell Dev Biol. 2024 Apr 24;12:1388745. doi: 10.3389/fcell.2024.1388745 (PMC11076789; doi:10.3389/fcell.2024.1388745)

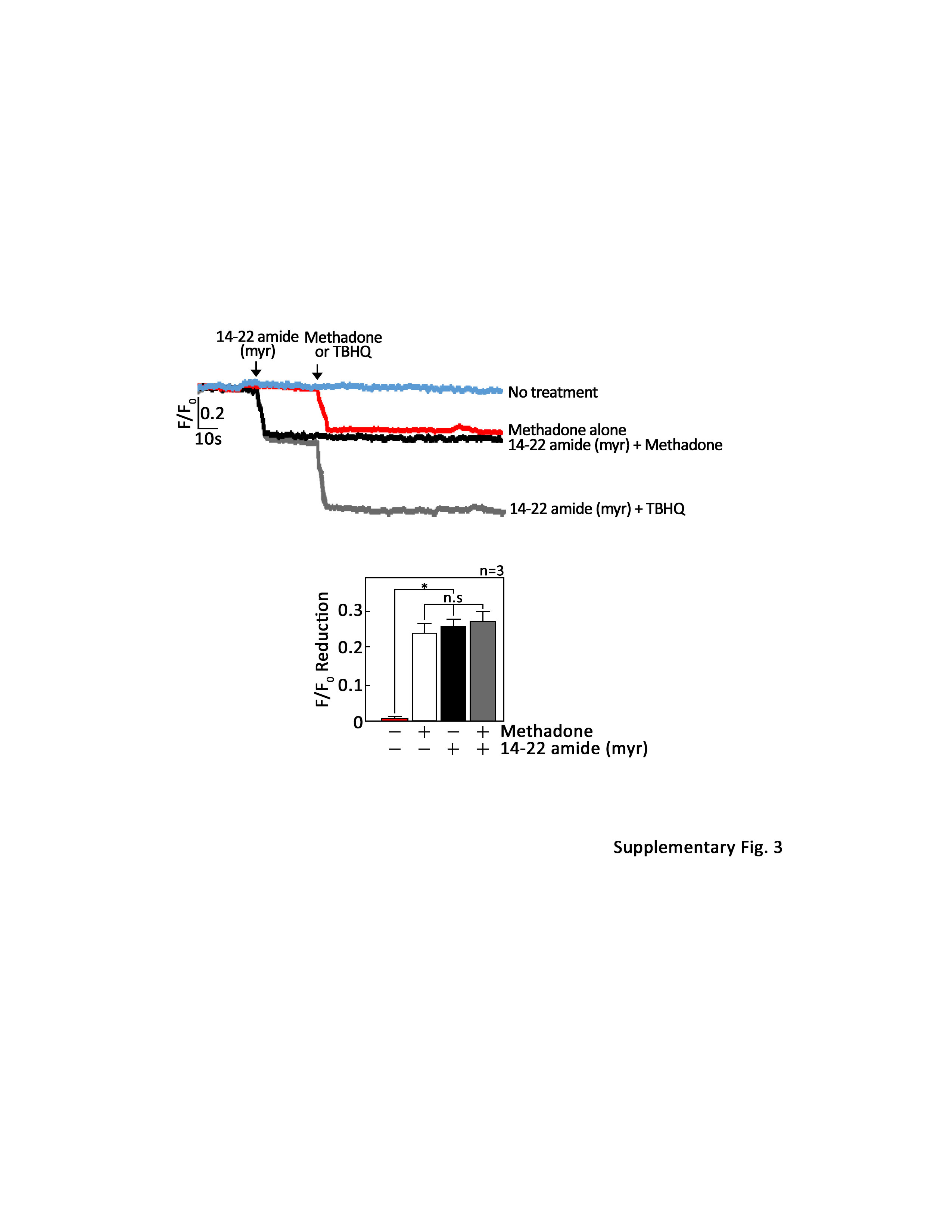

Supplement: Supplementary file 1 [file Image3.JPEG]

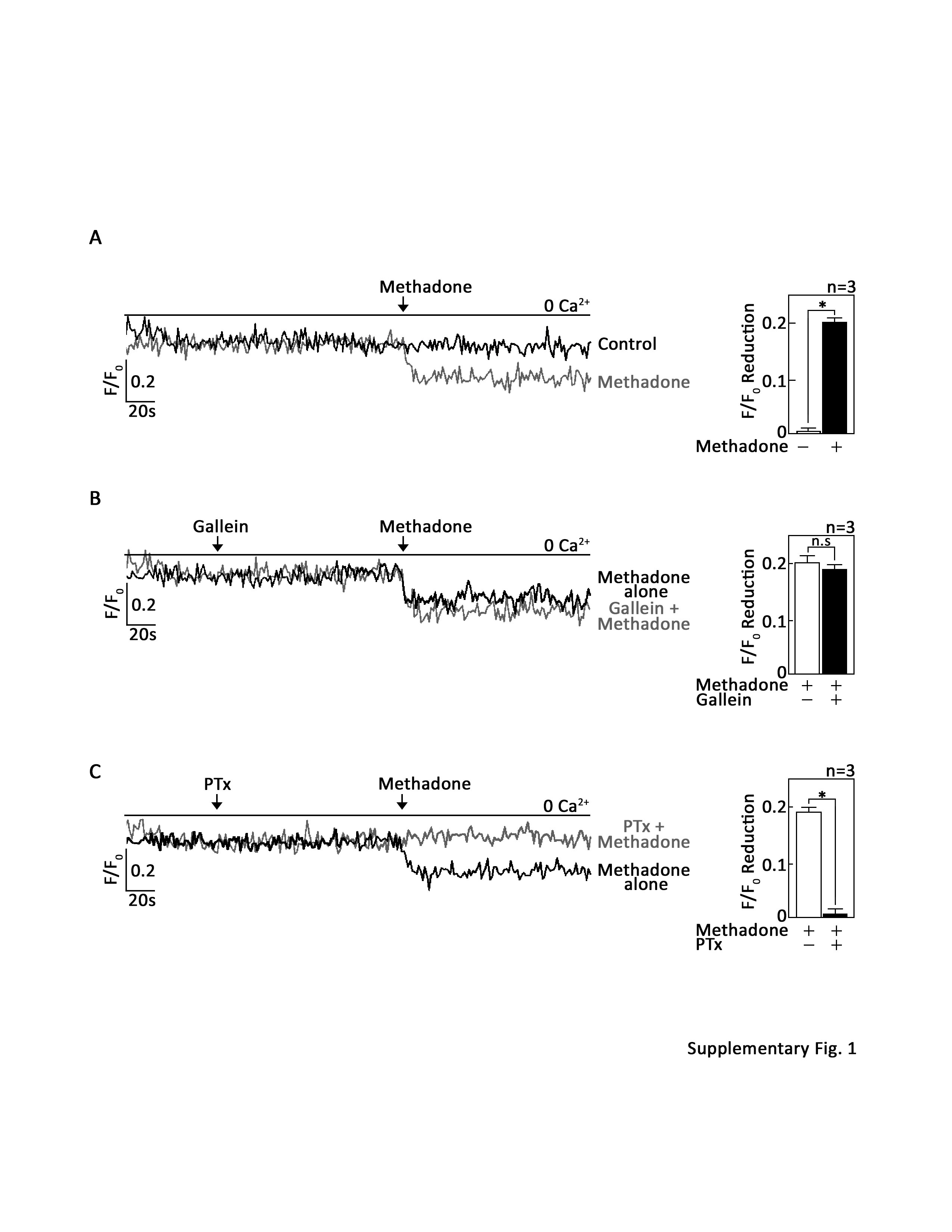

Supplement: Supplementary file 2 [file Image1.JPEG]

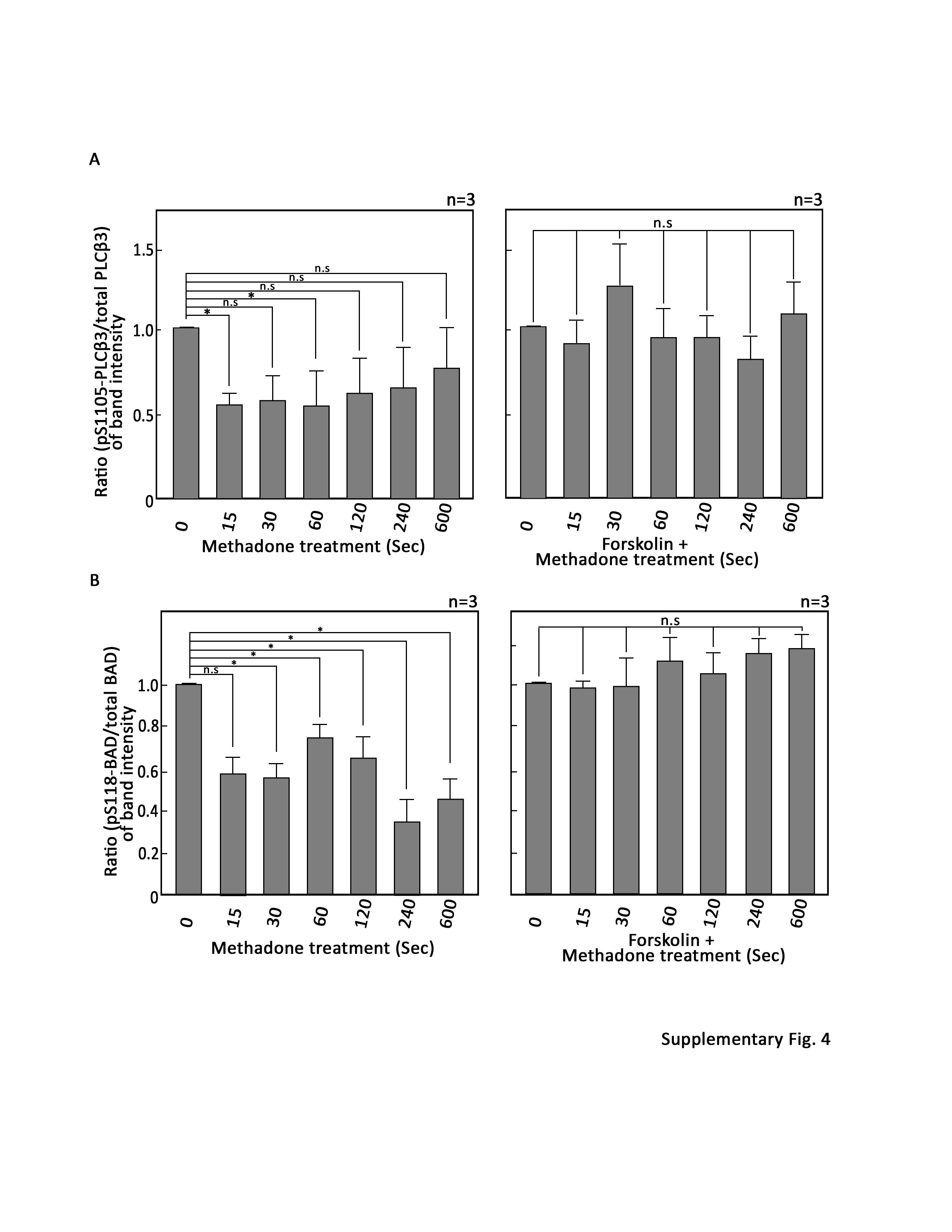

Supplement: Supplementary file 3 [file Image4.JPEG]

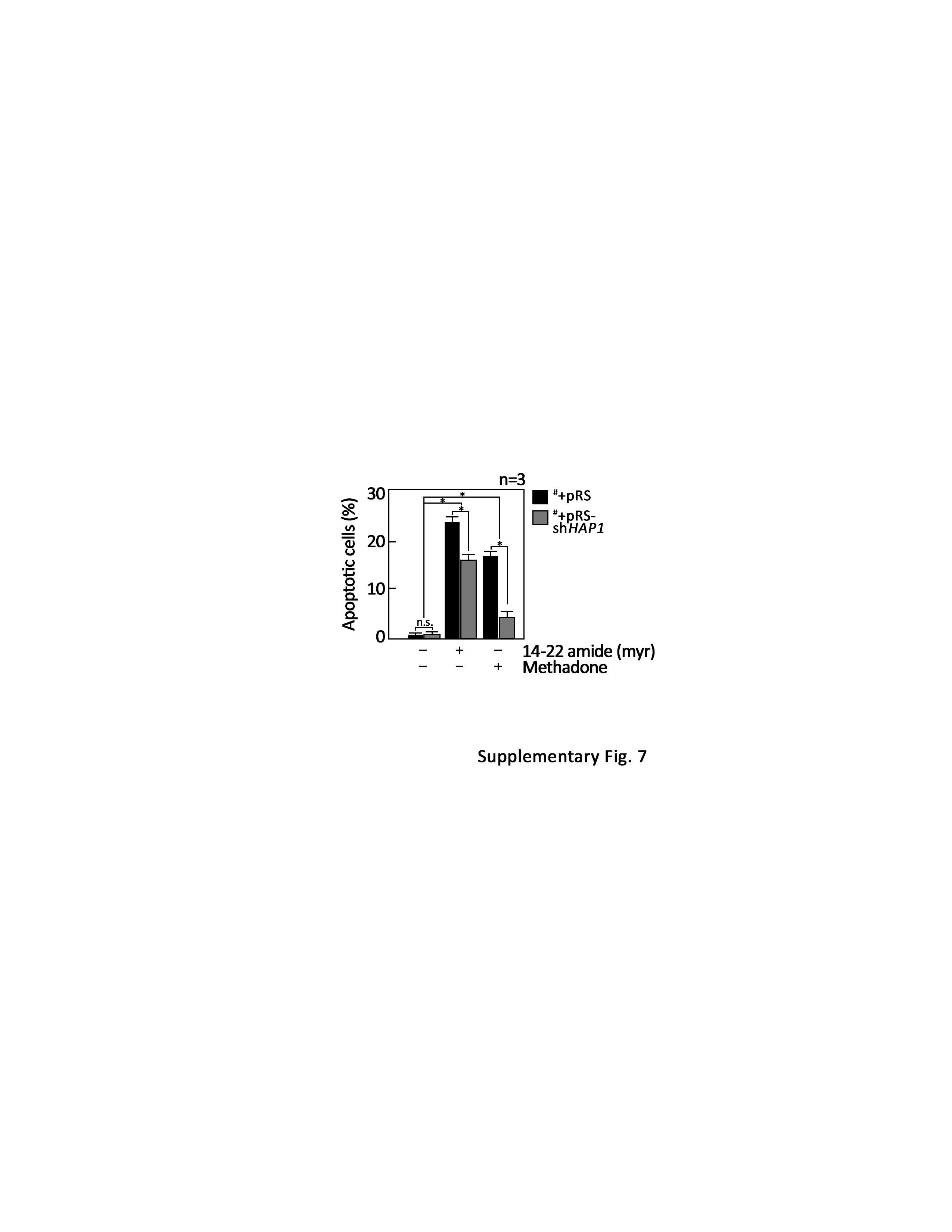

Supplement: Supplementary file 4 [file Image7.JPEG]

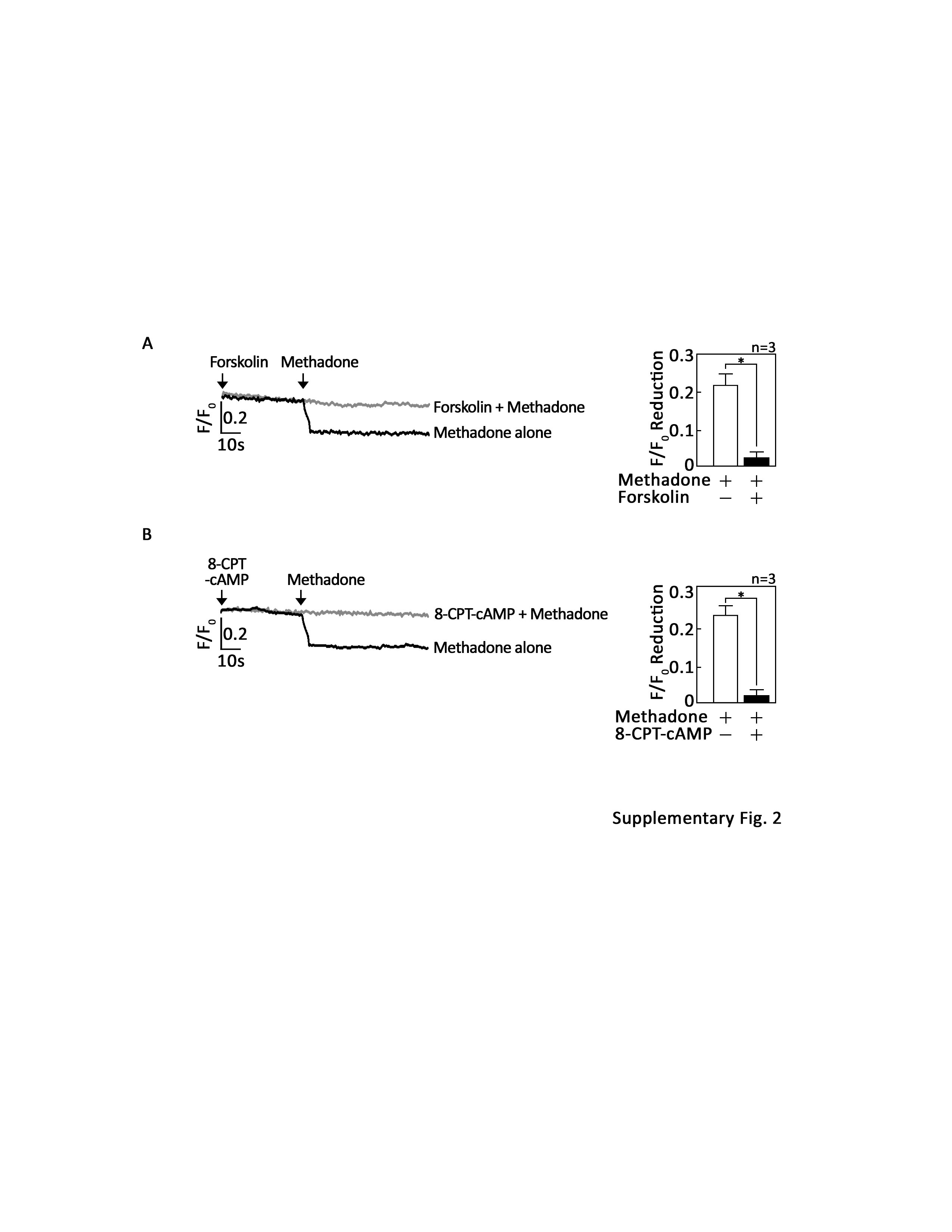

Supplement: Supplementary file 5 [file Image2.JPEG]

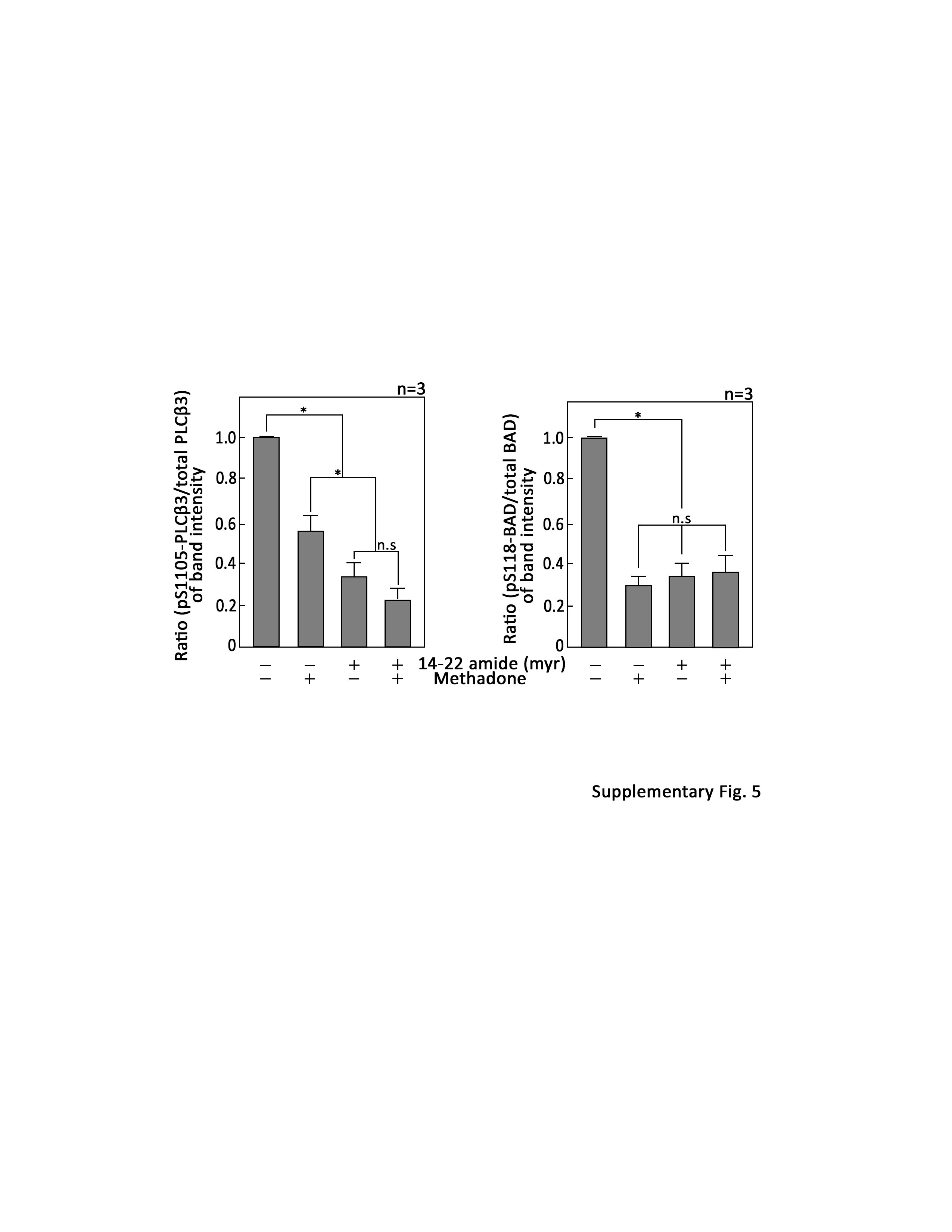

Supplement: Supplementary file 6 [file Image5.JPEG]

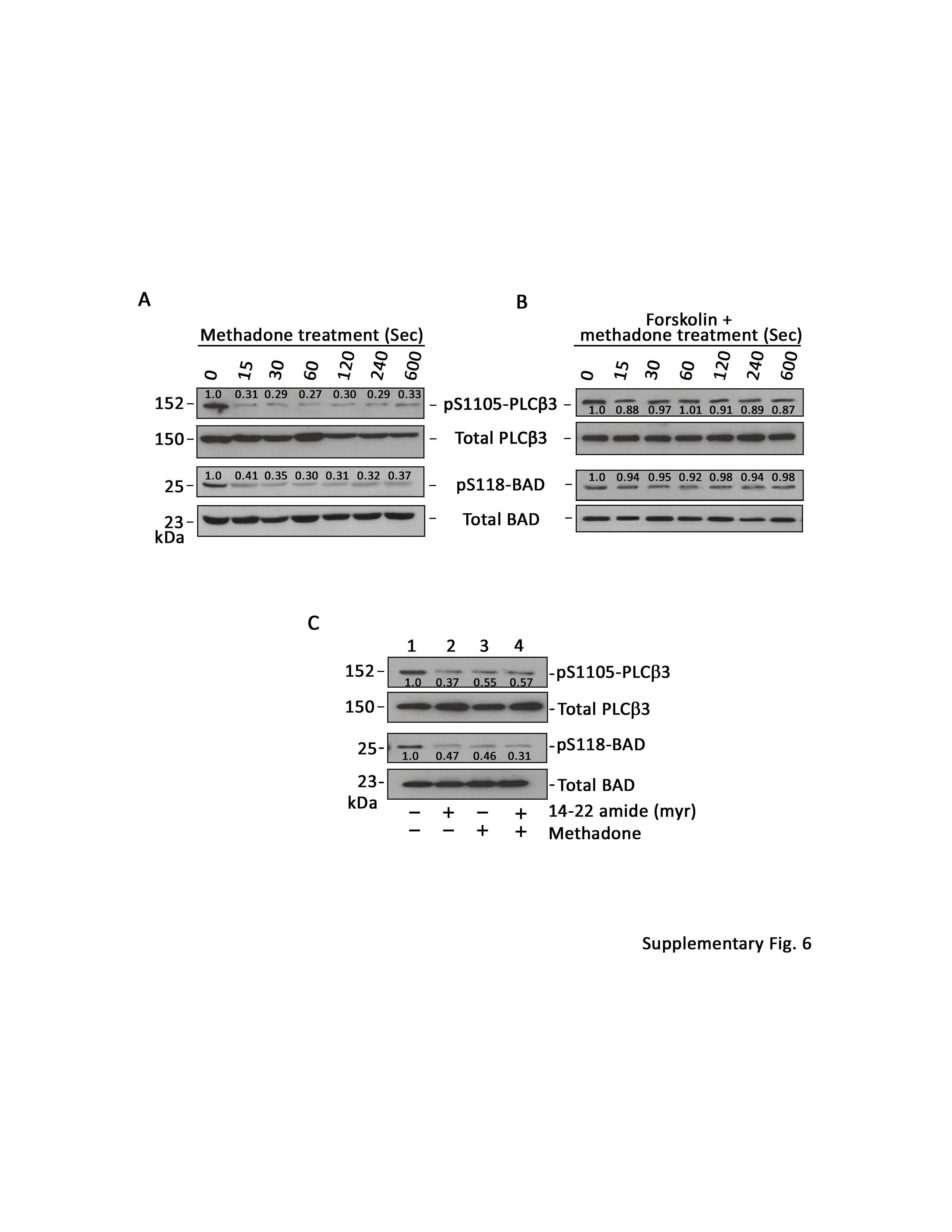

Supplement: Supplementary file 7 [file Image6.JPEG]
